# Proximal aortic stiffness in the paediatric adolescent population

**DOI:** 10.1186/1532-429X-13-S1-P389

**Published:** 2011-02-02

**Authors:** Adam J Nelson, James D Cameron, Adelene C Kaihui, Angelo Carbone, Scott R Willoughby, Cameron J van den Heuvel, James A Martin, Declan Kennedy, Stephen G Worthley, Matthew I Worthley

**Affiliations:** 1 University of Adelaide, Adelaide, Australia; 2 Monash Cardiovascular Research Centre, Melbourne, Australia

## Objective

The aim of this study was to assess whether arterial stiffness increases in the second decade of life.

## Background

Pathological studies have shown that atherosclerotic disease begins in early childhood. Furthermore, associations between risk factors and early atherosclerotic disease are evident in this population and therefore may be linked to future symptomatic coronary events.

While we have shown that ageing preferentially stiffens the proximal aorta in an adult population, it is uncertain whether this also occurs in an adolescent cohort.

## Methods

The evaluation of arterial stiffness was performed by gated magnetic resonance imaging (MRI, 1.5T Siemens Sonata) TrueFISP (fast imaging with steady state free precession) cine sequence with off-line analysis (Image-Pro Plus, MediaCyberkinetics, USA). Cross-sectional measurements of aortic areas (mm^2^) were undertaken at 3 locations: the ascending (AA), proximal descending (PDA) and distal descending aorta (DDA). Arterial stiffness was evaluated by aortic distensibility [(maximal aortic area - minimal aortic area)/(brachial pulse pressure X minimal aortic area)].

## Results

Ten paediatric (10.6±2.3 years) and ten young adults (20.3±0.5 years) underwent cardiovascular magnetic resonance assessment of arterial stiffness.

## Conclusions

This is the first study to demonstrate a similar pattern of heterogenous stiffening occurring as early as the 2nd decade. These results must be interpreted in the context of this study being cross sectional in nature and involving only a small number of subjects. Nonetheless these findings warrant further assessment through a prospective, longitudinal study to further evaluate this parameter as a potential unique early marker of future cardiovascular events.

**Table 1:** 

		Pediatrics	Young Adult	p
Brachial Pulse Pressure (mmHg)		38.3 ± 7.4	44.7 ± 9.1	NS

Aortic Distensibility (mmHg^-1^)
	AA	12.5 ± 4.6	7.8 ± 2.7	0.013

	PDA	9.6 ± 4.7	7.6 ± 2.5	NS

	DDA	12.9 ± 7.7	9.9 ± 3	NS

A significant difference (one-way ANOVA) is apparent in AA distensibility between the paediatric and young adult cohort (p<0.05) with no significant difference at the two more distal planes of assessment.

